# Gut hormone-based pharmacology: novel formulations and future possibilities for metabolic disease therapy

**DOI:** 10.1007/s00125-023-05929-0

**Published:** 2023-05-20

**Authors:** Matthias Tschöp, Ruben Nogueiras, Bo Ahrén

**Affiliations:** 1 Institute for Diabetes and Obesity, Helmholtz Zentrum, München, Germany; 2 Department of Physiology, University of Santiago de Compostela, Santiago de Compostela, Spain; 3 Department of Clinical Sciences Lund, Lund University, Lund, Sweden

**Keywords:** Diabetes, Double receptor agonists, GIP, GLP-1 receptor agonists, Glucagon, Obesity, Review, Triple receptor agonists

## Abstract

Glucagon-like peptide-1 (GLP-1) receptor agonists are established pharmaceutical therapies for the treatment of type 2 diabetes and obesity. They mimic the action of GLP-1 to reduce glucose levels through stimulation of insulin secretion and inhibition of glucagon secretion. They also reduce body weight by inducing satiety through central actions. The GLP-1 receptor agonists used clinically are based on exendin-4 and native GLP-1 and are available as formulations for daily or weekly s.c. or oral administration. GLP-1 receptor agonism is also achieved by inhibitors of dipeptidyl peptidase-4 (DPP-4), which prevent the inactivation of GLP-1 and glucose-dependent insulinotropic polypeptide (GIP), thereby prolonging their raised levels after meal ingestion. Other developments in GLP-1 receptor agonism include the formation of small orally available agonists and compounds with the potential to pharmaceutically stimulate GLP-1 secretion from the gut. In addition, GLP-1/glucagon and GLP-1/GIP dual receptor agonists and GLP-1/GIP/glucagon triple receptor agonists have shown the potential to reduce blood glucose levels and body weight through their effects on islets and peripheral tissues, improving beta cell function and stimulating energy expenditure. This review summarises developments in gut hormone-based therapies and presents the future outlook for their use in type 2 diabetes and obesity.

**Graphical Abstract:**

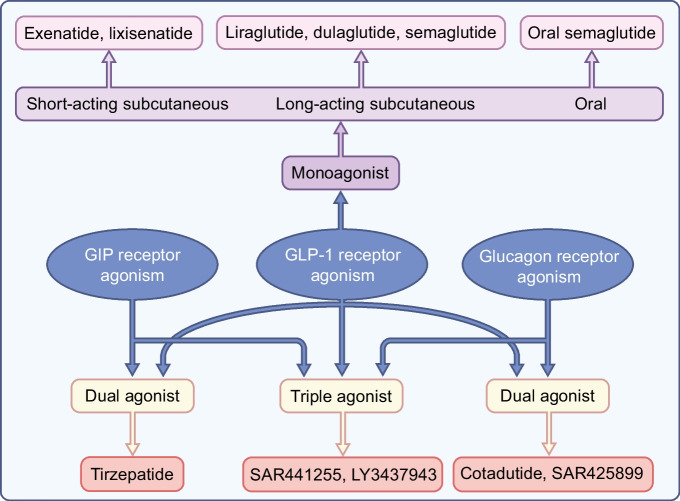

**Supplementary Information:**

The online version contains a slideset of the figures for download available at 10.1007/s00125-023-05929-0.

## Introduction

**Fig. 1:**
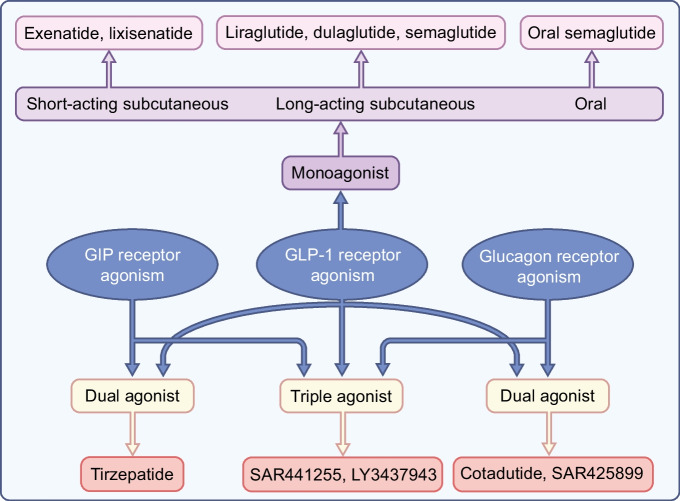
Schematic illustration of monoagonists, dual agonists and triple agonists based on GLP-1, GIP and glucagon receptor activation alone or in combination. This figure is available as part of a downloadable slideset

Professor Ernest Starling proposed the idea of using a gut hormone for the treatment of diabetes in London in the early 1900s and this idea was first tested by administering gut extracts to individuals with the disease [1]. However, this attempt failed and this approach did not prove successful until the introduction of the incretin hormone glucagon-like peptide-1 (GLP-1) as the basis for glucose-lowering therapeutics in type 2 diabetes. GLP-1-based therapy was developed in the 1990s and treatment with daily injectable GLP-1 receptor agonists and oral dipeptidyl peptidase-4 (DPP-4) inhibitors was introduced in the 2000s [2]. New formulations of GLP-1 receptor agonists with weekly injectable or oral administration were introduced in the 2010s [3]. More recently, novel formulations of double and triple gut hormone receptor agonists (involving glucose-dependent insulinotropic polypeptide [GIP] and glucagon) have been successfully developed [4], and other hormones, such as oestrogens and thyroid hormones, have also shown potential for use as therapies for the treatment of diabetes. Figure 1 illustrates schematically the formulations that have entered clinical development today. This review summarises the development of gut hormones as a successful therapy in type 2 diabetes and presents the outlook for the future.

## Glucagon-like peptide-1

**Fig. 2:**
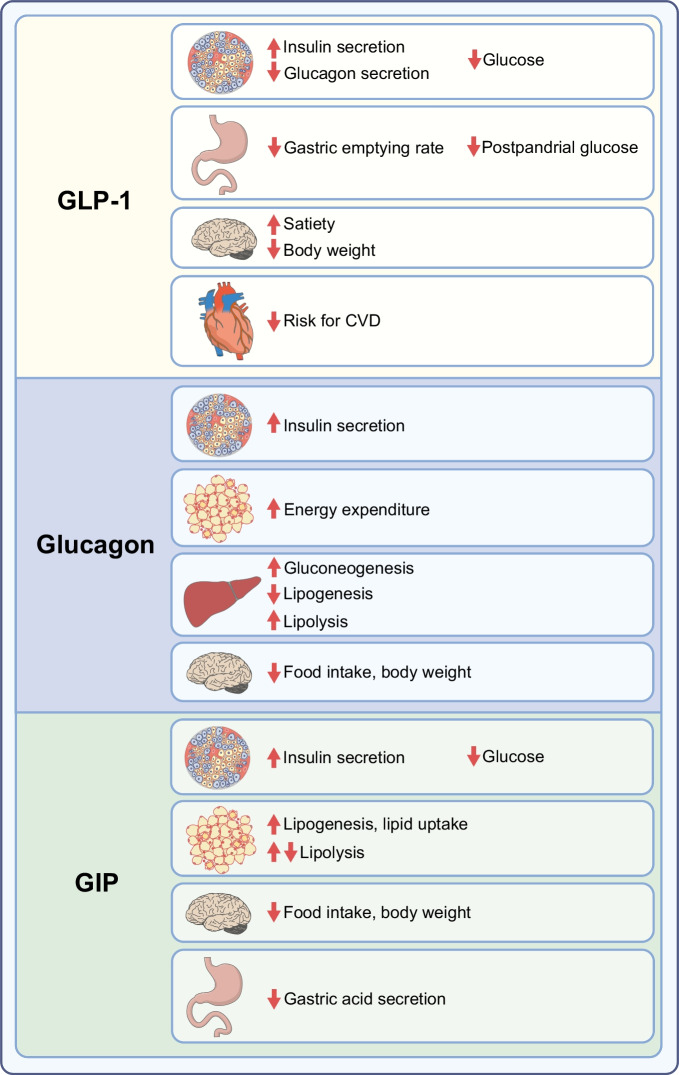
The clinically most relevant mechanisms of action of GLP-1, glucagon and GIP. These provide the main basis for the actions of GLP-1 receptor agonists and dual and triple receptor agonists in the therapy of type 2 diabetes and obesity. This figure is available as part of a downloadable slideset

GLP-1 is a 30 amino acid peptide that is produced by and released from intestinal L cells after meal ingestion and which in turn stimulates insulin secretion [5]. The pharmacological effects of GLP-1-based therapy are achieved by activating the GLP-1 receptor, which is a class B, G protein-coupled receptor with 463 amino acids. The receptor is a glycoprotein with an N-terminal extracellular signal peptide and, like all G protein-coupled receptors, a seven-transmembrane helix domain [6]. As recently reviewed in detail [5], activation of GLP-1 receptors leads to the formation of cAMP from ATP. The rise in cAMP leads, in turn, to activation of protein kinase A (PKA) and enhanced signalling via exchange proteins directly activated by cAMP (EPACs), in particular EPAC2. The increase in PKA activity also leads to closure of ATP-sensitive potassium channels, which in turn causes depolarisation of the cell membrane and opening of voltage-dependent calcium channels, resulting in uptake of calcium and stimulation of the exocytosis of secretory granules and thus secretion of insulin. Furthermore, the activation of EPAC2 stimulates the release of calcium from the endoplasmic reticulum, which raises intracellular calcium levels and promotes exocytosis. GLP-1 receptor activation also inhibits beta cell apoptosis. Other effects of activation of GLP-1 receptors include inhibition of glucagon secretion combined with inhibition of gastric emptying, suppression of appetite and cardioprotective effects [5]. Whether GLP-1 increases energy expenditure has also been examined. However, although there are indications that this is the case in animal models, through the regulation of brown adipose tissue activity, particularly in diet-induced obese mice [7], there is no convincing evidence that this occurs in humans [5]. Overall, therefore, the main effects of GLP-1 are a reduction of fasting and postprandial blood glucose levels, lowering of body weight and protection from CVD, which are all therapeutic targets in type 2 diabetes (Fig. 2).

A challenge during the early development of GLP-1-based therapy was its short half-life in the circulation (approximately 2 min after i.v. administration and 1.5 h after s.c. administration) [8]. Therefore, the initial demonstrations of a glucose-lowering effect of GLP-1 used continuous i.v. or s.c. infusions [9, 10]. To harness the promising effects of GLP-1 for the development of a clinically useful drug, the short duration of action needed to be overcome.

## Formulations of GLP-1 with extended duration

**Fig. 3:**
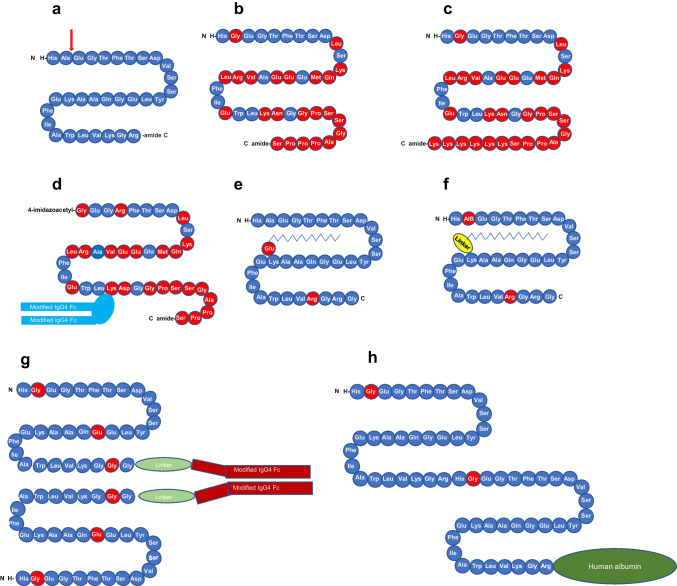
Structures of formulations of GLP-1 receptor agonists [14, 15, 19–21, 26]. (**a**) GLP-1, (**b**) exenatide, (**c**) lixisenatide, (**d**) efpeglenatide, (**e**) liraglutide, (**f**) semaglutide, (**g**) dulaglutide, (**h**) albiglutide. Amino acids are illustrated in circles; red circles show amino acids that are different from those in GLP-1. The red arrow in (**a**) illustrates the site of DPP-4 inactivation. This figure is available as part of a downloadable slideset

The enzyme DPP-4 is responsible for the rapid inactivation of GLP-1, although some GLP-1 is also removed from the circulation by renal clearance and by the enzyme neutral endopeptidase [11]. DPP-4 is a proteolytic glycoprotein that removes the two N-terminal amino acids of small peptides when the second amino acid from the N-terminal side is alanine or proline [12]. In GLP-1, this amino acid is alanine (Fig. 3a) and therefore the N-terminal dipeptide is removed by DPP-4. This inactivates GLP-1 as an intact N-terminal end is required for binding to its receptor [13]. One strategy for extending the half-life of GLP-1 was therefore to develop formulations of DPP-4-resistant GLP-1 receptor activators; this was usually achieved by replacing alanine in the second position with another amino acid other than proline. Other strategies have involved producing formulations with increased binding to albumin through a fatty acid linker [14]; creating big complexes by fusing GLP-1 with molecules such as albumin [15] or immunoglobulins [16]; and forming microspheres by fusion of a GLP-1 receptor agonist with poly(d,l-lactide-*co*-glycolic acid) [17]. These larger molecules show reduced renal clearance as well as being DPP-4 resistant.

### Exendin-4-based GLP-1 receptor agonists

The first GLP-1 receptor agonist to be used clinically was the recombinant form of the peptide exendin-4, called exenatide. Exendin-4 was isolated from venom produced by the perimandibular glands of the Gila monster (*Heloderma suspectum*) [18]. This peptide is a GLP-1 receptor agonist with a 53% similarity to GLP-1 (Fig. 3b). Exenatide is resistant to DPP-4 inactivation and has a half-life of approximately 2.4 h after s.c. administration. It was developed as a twice-daily therapy [19] and was approved by the US Food and Drug Administration (FDA) in 2005 and by the European Medicines Agency (EMA) in 2006. It effectively reduces postprandial glucose levels owing to a marked inhibitory effect on gastric emptying, and it also reduces fasting glucose levels and body weight; however, its effects last for only a few hours. Therefore, a formulation of exenatide based on microsphere technology using fusion with poly(d,l-lactide-*co*-glycolic acid) was developed [17]. This form is administered once weekly and was approved by the FDA and EMA in 2011.

The second exendin-4-derived GLP-1 receptor agonist to be used clinically was lixisenatide. Lixisenatide is a modified exendin-4 in which proline has been deleted from the C-terminal end and six lysines have been added (Fig. 3c). These modifications increased the half-life to approximately 3 h after s.c. administration, allowing lixisenatide to be developed as a once-daily injectable [20]. It was approved by the FDA and EMA in 2013. It is particularly effective at inhibiting gastric emptying and therefore reducing postprandial glucose levels [3]. However, as the effects do not persist over an entire 24 h period, its efficacy at lowering blood glucose levels and reducing body weight is lower than that of GLP-1 receptor agonists with a longer duration of action [3].

Recently, an exendin 4-based GLP-1 receptor agonist with a long duration of action, allowing once-weekly administration, has been developed. Efpeglenatide is a modified exendin-4 that, through a mini-polyethylene glycol linker, has been conjugated to an F_C_ fragment of human immunoglobulin 4 (Fig. 3d [21]). This compound has a half-life of approximately 5–7 days after s.c. administration owing to both DPP-4 resistance and a delay in kidney removal because of its large size. It has gone through a development programme, including a cardiovascular outcomes trial, which have shown beneficial effects, but has not yet been approved [22].

### GLP-1 analogues as GLP-1 receptor activators

Most GLP-1 receptor agonists in clinical use are GLP-1 analogues, that is, peptide formulations based on native GLP-1 and showing a high degree of similarity to native GLP-1.

The first GLP-1 analogue to be developed was liraglutide (Fig. 3e). In liraglutide, palmitate, which is a C-16 fatty acid, is coupled to amino acid 20 of GLP-1 through a gamma-glutamate linker [14]. This modification reversibly increases binding to albumin, which results in protection of liraglutide from degradation by DPP-4 and reduces renal clearance. Liraglutide has a half-life of approximately 13 h after s.c. administration, which allows once-daily administration with activity throughout the 24 h period. Liraglutide was therefore the first long-acting GLP-1 receptor agonist with continuous efficacy over a 24 h period. It was approved by the FDA and EMA for clinical use in 2009 [23] and was also shown to have positive outcomes in a cardiovascular outcomes trial [22].

A similar technique was used for the development of semaglutide. Stearic diacid, which is a C-18 fatty acid, was added through a linker to amino acid 20 of GLP-1 and, in addition, the second amino acid in GLP-1 was changed from alanine to alpha-aminoisobutyric acid (Fig. 3f [14]). Semaglutide was shown to be effective at reducing glucose levels and body weight with once-weekly s.c. administration [24]. It was approved by the FDA in 2017 and by the EMA in 2018. It was also shown to have positive outcomes in a cardiovascular outcomes trial [22].

Another strategy to prolong the duration of action of GLP-1 receptor agonists is to covalently fuse them to a larger protein. In dulaglutide, two modified DPP-4-resistant GLP-1 molecules are each fused to a modified F_C_ fragment of human immunoglobulin 4 via a small peptide (Fig. 3g [25]). A similar modification was performed in albiglutide; in this case, two sequential DPP-4-resistant GLP-1 molecules are fused with human albumin (Fig. 3h [15, 26]). Both dulaglutide and albiglutide have a long duration of action, which allows for once-weekly administration. They have both gone through extensive clinical programmes, including cardiovascular outcomes trials, and have shown beneficial effects [22, 26, 27]. They were approved for therapy by the FDA and EMA in 2014, although albiglutide was withdrawn from the market in 2018 for economic reasons.

### Orally available GLP-1 analogues

A recent development has been the formulation of GLP-1 receptor agonists for oral administration. To date, the only oral GLP-1 receptor agonist available for clinical use is oral semaglutide. The formulation of oral semaglutide was achieved by coupling semaglutide to sodium-*N*-[8-(2-hydroxybezoyl)-amino]caprylate (SNAC), which functions as an absorption enhancer. SNAC raises the local pH in the stomach, which leads to higher solubility of semaglutide and protection from degradation by gastric acid. When the SNAC–semaglutide complex reaches the gastric epithelium, the lipophilic SNAC enters the cell membrane, which affects its fluidity, leading to rapid transcellular absorption of semaglutide [14, 28]. However, absorption is still low and ingestion of a 14 mg tablet of semaglutide is required to achieve comparable plasma levels to those achieved with 1 mg of the s.c. form [29]. Oral semaglutide is taken once daily and reduces HbA_1c_ levels to a significantly higher extent than DPP-4 inhibition or sodium–glucose cotransporter 2 (SGLT-2) inhibition and to a similar extent as liraglutide, whereas body weight is reduced by a significantly higher extent than by DPP-4 inhibition and liraglutide and to a similar extent as SGLT-2 inhibition [30, 31]. It was approved by the FDA in 2019 and by the EMA in 2020.

### Differentiation between GLP-1 receptor agonists

The clinically developed GLP-1 receptor agonists have all been shown to have glucose-lowering effects. Of most importance are the stimulation of beta cell function, reduction of glucagon secretion and delay in gastric emptying, which together lower fasting and postprandial glucose and HbA_1c_ levels; the induction of satiety with reduction in body weight; and the beneficial effects on cardiovascular risk, as evident from the cardiovascular outcomes trials (Fig. 2). The longer acting GLP-1 receptor agonists are more effective than the shorter acting forms at reducing HbA_1c_ and fasting glucose levels, whereas the shorter acting forms are more effective at reducing postprandial glucose levels [3]. The latter differentiation depends on the sustained ability of short-acting GLP-1 receptor agonists to delay gastric emptying by inhibiting gastric motility, as tachyphylaxia of this effect appears after long-term GLP-1 receptor agonism [3]. Furthermore, the smaller longer acting forms are more effective than the larger longer acting forms at reducing body weight [3], which may be due to the smaller forms more readily passing the blood–brain barrier. All GLP-1 receptor agonists are safe in terms of cardiovascular outcomes, and liraglutide, semaglutide, albiglutide, dulaglutide and efpeglenatide have been shown to have cardiovascular benefits in cardiovascular outcomes trials [22]. GLP-1 receptor agonists are associated with a minimal risk of hypoglycaemia because of the glucose dependency of the islet effects [32]. Furthermore, adverse events are rare, except for nausea and vomiting during the initial phase of therapy, and are similar between the GLP-1 receptor agonists [3]. It may therefore be concluded that, as glucose-lowering therapy, semaglutide (both s.c. and oral forms) and dulaglutide have an advantage over the other GLP-1 receptor agonists in terms of lowering glucose; semaglutide (both s.c. and oral forms) has an advantage in terms of reducing body weight; and liraglutide, s.c. semaglutide and dulaglutide have beneficial effects on cardiovascular outcomes. This provides the background to the recent recommendations on the management of hyperglycaemia in type 2 diabetes by the EASD and ADA [33].

## Alternative strategies to harness the glucose-lowering action of GLP-1

Besides the production of formulations of GLP-1 receptor agonists with longer durations of action, other strategies for harnessing the glucose-lowering actions of GLP-1 have been developed. The most successful of these, and to date the only one that has reached the clinic, is DPP-4 inhibition.

### DPP-4 inhibitors (gliptins)

Shortly after the demonstration that the enzyme DPP-4 is responsible for the rapid inactivation of GLP-1, the idea of inhibiting DPP-4 to prolong the duration of action of GLP-1 (and glucose-dependent insulinotropic polypeptide [GIP]) was put forward (reviewed in [34]). DPP-4 inhibition increases intact and active GLP-1 (and GIP) levels after a meal and the levels remain elevated until the next meal. This results in effects secondary to both GLP-1 and GIP receptor activation, such as increased beta cell function and inhibition of glucagon secretion [35]. The levels of GLP-1 achieved are lower than the corresponding levels seen after administration of GLP-1 receptor agonists. Therefore, although DPP-4 inhibition reduces both fasting and postprandial glucose and HbA_1c_ levels, the effects are weaker than for GLP-1 receptor agonists, and they do not exhibit a body weight reduction effect, although they are body weight neutral. The first demonstration that DPP-4 inhibition reduces blood glucose levels in type 2 diabetes was published in 2002 [36]. The first DPP-4 inhibitor, sitagliptin, was approved by the FDA in 2006 and by the EMA in 2007; this was followed by approval of vildagliptin, alogliptin, saxagliptin and lingaliptin [37]. Several other small molecules that inhibit DPP-4 have since been developed, such as gemagliptin, anagliptin and teneligliptin. DPP-4 inhibitors reduce HbA_1c_ levels and have a low risk of hypoglycaemia or other adverse events [38], except for a potential risk of hospitalisation for heart failure in the case of saxagliptin [38]. They were also shown to be safe in large cardiovascular outcomes trials but were not found to have cardioprotective effects [39]. Today, their use has increased worldwide and they have a major role in primary care as glucose-lowering therapy among older people [40].

### Non-peptide small GLP-1 receptor agonists

Recently, novel insights into the binding characteristics of the GLP-1 receptor have allowed the development of non-peptide GLP-1 receptor activators [41, 42]. These small molecules bind to the receptor, stimulate insulin secretion and cAMP production in a glucose-dependent mechanism and also lower glucose in experimental models of diabetes in animals. A Phase I trial of the small non-peptide GLP-1 receptor agonist danuglipron in 98 patients with type 2 diabetes found that it had a similar glucose-lowering ability as the clinically used GLP-1 receptor agonists and was well tolerated during the 4 week study period [43].

### L cell secretagogues

Stimulation of GLP-1 secretion from enteroendocrine L cells is another approach that may be able to realise the therapeutic potential of GLP-1, perhaps in combination with DPP-4 inhibition to prevent the inactivation of the GLP-1 released. The regulation of GLP-1 secretion both in humans [44] and at the cellular level [45] has been studied but, except for the finding that ingestion of whey protein as a preload 30 min before a meal augments the GLP-1 response, with clinical benefits for type 2 diabetes [46], it has been difficult to translate the knowledge gained into clinically relevant formulations. A trial of encapsulated glutamine, for example, failed to increase GLP-1 secretion in healthy participants or those with type 2 diabetes [47]. Newer approaches, such as activation of the Takeda G protein-coupled receptor 5 (TGR5; bile acid receptor) in L cells have shown more promise in experimental systems [48], but the results have not yet been translated to humans.

## Glucagon

Glucagon is processed from its precursor, proglucagon, by prohormone convertase 2 and secreted from pancreatic alpha cells. The role of glucagon in maintaining glucose homeostasis by increasing hepatic gluconeogenesis and glycogenolysis in response to low glucose levels has been exhaustively studied. The sustained action of glucagon causes hyperglycaemia, and glucose-mediated inhibition of glucagon secretion is impaired in patients with type 2 diabetes (see comprehensive reviews in [49, 50]). Glucagon exerts its actions via the glucagon receptor, a seven-transmembrane receptor coupled to Gα_s_ and G_q_ proteins. The glucagon receptor is primarily expressed in the liver but also in the central nervous system, kidney, gastrointestinal tract and pancreas [49, 50].

In addition to its hyperglycaemic action, glucagon has been reported to activate lipolysis and inhibit lipogenesis in the liver. For instance, the administration of glucagon receptor antagonists increased hepatic fat content and plasma concentrations of LDL-cholesterol in people with type 2 diabetes [51]. Furthermore, leptin receptor-deficient mice, a mouse model of obesity and diabetes, and people with endogenous glucagon deficiency (pancreatectomised individuals) treated with glucagon antisense oligonucleotide have increased hepatic fat [52, 53]. In addition, *Gcgr*^−/−^ mice developed steatosis when fed a high fat diet [54]. These data suggest that inhibition of glucagon receptor signalling results in hepatic lipid accumulation. Consistent with this, the acute administration of glucagon decreased NEFA and triacylglycerol plasma concentrations and reduced hepatic triglyceride content in wild-type mice [54].

Glucagon has also been shown to promote satiety and to increase energy expenditure in both rodents and humans. The satiety effect of glucagon was blocked after disconnection of the hepatic branch of the abdominal vagus nerve [55]. At a central level, the anorectic effect of glucagon is mediated by a hypothalamic pathway involving a PKA/Ca^2+^–calmodulin-dependent protein kinase kinase β (CaMKKβ)/AMP-activated protein kinase (AMPK)-dependent mechanism [56]. The ability of glucagon to increase energy expenditure was first demonstrated in rats in 1960 [57] and was subsequently confirmed in different species including humans [58], although one study reported that glucagon infusion over 72 h did not increase energy expenditure in healthy individuals with overweight or obesity [59]. In clinical studies, the stimulatory effect of glucagon on energy expenditure is heterogeneous depending on feeding status (preprandial vs postprandial) and the mechanism by which this occurs is not known. Rodents can increase their energy expenditure via activation of brown adipose tissue (BAT). However, glucagon can stimulate energy expenditure in species with little BAT (adult dogs) or no BAT (pigs) activity. Therefore, it is accepted that glucagon may affect energy expenditure via BAT-independent mechanisms (for review see [60, 61]). Circulating fibroblast growth factor 21 (FGF21) is also implicated in glucagon-induced energy expenditure, as mice lacking FGF21 are protected from this effect [62]. Whether this mechanism occurs in other species remains to be investigated.

Therefore, despite its hyperglycaemic action, glucagon triggers lipid catabolism and energy expenditure and reduces food intake (Fig. 2). These features support the rationale for using glucagon in combination with other gut hormones as described below.

## Glucose-dependent insulinotropic polypeptide

**Fig. 4:**
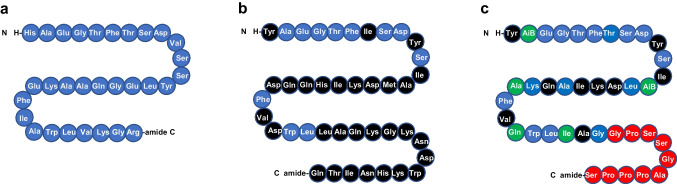
Structures of (**a**) GLP-1, (**b**) GIP and (**c**) tirzepatide [19, 88, 90]. Amino acids are illustrated in circles; blue circles show amino acids that are identical to those in GLP-1; black circles show amino acids that are present in GIP and tirzepatide but not in GLP-1; red circles show amino acids that are identical in tirzepatide and exenatide (see Fig. 3b); and green circles show amino acids that are present in tirzepatide but not in GLP-1, GIP or exenatide. AiB, aminoisobutyric acid. This figure is available as part of a downloadable slideset

GIP is a 42 amino acid protein (Fig. 4b) that is secreted from K cells in the mucosa of the duodenum and jejunum and which also exerts its actions through a G protein-coupled receptor. GIP was originally reported to inhibit gastric acid secretion and was thus named gastric inhibitory polypeptide. As GIP became established as an incretin hormone potentiating insulin release from beta cells in a glucose-dependent manner, it was renamed glucose-dependent insulinotropic polypeptide. The insulinotropic effect of GIP and GLP-1 is additive in healthy humans but is impaired in people with type 2 diabetes [61]. GIP also exhibits protective effects on the survival of pancreatic beta cells [63].

GIP has also been shown to have actions beyond its effect on insulin secretion (Fig. 2). It has been reported to modulate fatty acid metabolism but its role in this respect remains unclear. Some studies have shown that it stimulates lipogenesis and lipid uptake in adipose tissue and reduces lipolysis [64]. Consistent with this, whole-body GIP receptor deficiency and GIP deficiency protect against obesity induced by long-term high fat feeding in mice [65, 66]. However, other studies have reported that GIP may have lipolytic activity [67] and that GIP-overexpressing transgenic mice exhibit resistance to high fat diet feeding [68]. Furthermore, the activation of the GIP receptor also improved glucose metabolism in diet-induced obese mice without changing body weight or fat mass [69]. In line with this, activation of the GIP receptor in the hypothalamus (the brain GIP receptor is located in the hypothalamus and hindbrain) decreased food intake in diet-induced obese mice [70, 71]. These preclinical results were supported by genome-wide association studies, which have identified variants with reduced activity at the human GIP receptor locus that are associated with reduced BMI [72]. More recent studies have shown that GIP analogues lead to weight loss, especially in combination with GLP-1 receptor agonists (reviewed in [71]). One such study showed that chronic daily administration of GIP analogues decreased food intake and resulted in body weight reduction in diet-induced obese mice without alterations in energy expenditure [73].

A recent elegant study also found that brain-*Gipr* knockout mice and humanised (*h*)*GIPR* knock-in mice with brain-*hGIPR* deletion showed decreased body weight and improved glucose metabolism [70]. In addition, acute central and peripheral administration of acyl-GIP increased c-Fos neuronal activity in hypothalamic feeding centres and decreased body weight and food intake [70]. Thus, it appears that brain GIP receptor signalling plays a key role in the regulation of energy balance. These findings also indicate that, at least at the central level, agonism of the GIP receptor exerts a catabolic action, suggesting that the reduction in body weight found in other studies after the administration of GIP receptor antagonists was not mediated by the brain.

Additional studies have demonstrated that combining GIP and GLP-1 receptor agonists is more effective at reducing body weight than using either individually. For instance, the co-administration of acyl-GIP and acyl-GLP-1 decreased body weight, food intake and fat mass to a greater degree than administration of either of the agonists alone [74]. Indeed, there are now several dual GIP/GLP-1 receptor agonists in clinical development, including tirzepatide, which was approved by the FDA and EMA for the treatment of type 2 diabetes in 2022.

## Dual/triple peptides for the treatment of obesity and diabetes

### Dual glucagon/GLP-1 receptor co-agonists

The use of glucagon in patients with type 2 diabetes might seem illogical because of its action of promoting hyperglycaemia. However, as described earlier, its favourable effects on lipolysis and energy expenditure while reducing food intake make it an attractive option, especially if combined with the insulinotropic action of GLP-1. Moreover, previous studies have indicated that oxyntomodulin, which binds to both the glucagon receptor and the GLP-1 receptor, increases energy expenditure and decreases energy intake [75]. In 2009, the first unimolecular gut hormone receptor co-agonist, a glucagon/GLP-1 receptor co-agonist, was discovered and validated in preclinical models [76]. Weekly administration of this molecule normalised glucose tolerance and adiposity in diet-induced obese mice. Body weight reduction was achieved by the reduction of food intake and increased energy expenditure [76]. These discoveries were later confirmed using a similar glucagon/long-acting GLP-1 dual receptor agonist; this peptide resulted in weight loss and had lipid-lowering and glucose-lowering effects in mice with diet-induced obesity, which were superior to the effects seen with a GLP-1 receptor-selective agonist [77].

Since the design and characterisation of these two compounds, other GLP-1/glucagon receptor co-agonists have been generated and the results from some clinical trials have been published. SAR425899 and MEDI0382 (also named cotadutide) have undergone Phase II clinical trials [78, 79]. These co-agonists have been shown to reduce glucose levels and body weight in people with overweight/obesity and type 2 diabetes, with nausea and vomiting the most frequently detected side effects (reviewed in [80, 81]). For instance, in a randomised, placebo-controlled, double-blind Phase I study of ascending single doses of cotadutide in healthy individuals with overweight, there were dose-dependent improvements in glucose excursions post meal within 24 h and food intake reduction after a single dose of 100 μg [82]. In addition, cotadutide was shown to be safe and, in common with the GLP-1 analogues, was associated with dose-dependent gastrointestinal adverse events, especially nausea and vomiting [82]. In a combined multiple ascending dose and Phase IIa study in people with type 2 diabetes over 41 days, daily doses of cotadutide up to 200 μg improved fasting and postprandial glucose levels and reduced body weight compared with placebo [79]. In a separate follow-up Phase IIa study over 49 days, the mechanism of improved glucose metabolism was shown to be a combination of enhanced insulin secretion and delayed gastric emptying [83]. While SAR425899 has been discontinued, cotadutide is being evaluated in participants with non-cirrhotic non-alcoholic steatohepatitis with fibrosis because of its beneficial effects in animal studies [84].

### Dual GIP/GLP-1 receptor co-agonists

The discovery of the first GIP/GLP-1 receptor co-agonist was reported in 2013 [74]. Its glucose-lowering and insulinotropic efficacy was superior to that of selective GLP-1 receptor agonists in diet-induced obese and leptin receptor-deficient mice, as well as in monkeys and humans [74]. The subsequent development of other GIP/GLP-1 receptor co-agonists confirmed these initial discoveries and the superior action profile of this drug class in obese rodents [85–87]. One of the more recent representatives of this drug class, and the one that has been shown to exert a superior effect to GLP-1 receptor agonists in humans, is LY3298176/tirzepatide, a synthetic linear peptide molecule containing 39 amino acids (Fig. 4c [88]). Tirzepatide binds with a higher affinity to the GIP receptor than the GLP-1 receptor. In receptor binding studies, the affinity of tirzepatide was comparable to that of native GIP for the GIP receptor and approximately fivefold weaker than that of native GLP-1 for the GLP-1 receptor [88]. In HEK293 cells expressing human GIP receptor or GLP-1 receptor, tirzepatide potently stimulated cAMP accumulation by both receptors, and the potency of tirzepatide was similar to that of native GIP and approximately 13-fold weaker than that of GLP-1 in these assays [88]. However, despite affinity, albeit lower, of tirzepatide for the GLP-1 receptor, tirzepatide had no effect on body weight, food intake, fasting insulin, endogenous glucose production and insulin-stimulated glucose uptake in skeletal muscle and subcutaneous white adipose tissue of GLP-1 receptor knockout mice [89].

Tirzepatide was approved by the FDA and EMA for the treatment of type 2 diabetes in 2022. Phase III trials were carried out as part of the SURPASS programme to investigate its therapeutic effectiveness (glycaemic control and body weight reduction) and safety/tolerability for the treatment of patients with type 2 diabetes [90, 91]. Tirzepatide treatment was administered at three final doses (5, 10, 15 mg per week), with treatment initiated at 2.5 mg and the dose escalated every 4 weeks. These trials showed that tirzepatide reduced fasting plasma glucose and HbA_1c_ levels not only compared with placebo but also compared with the insulins degludec and glargine or semaglutide [90–92]. Interestingly, tirzepatide also markedly reduced body weight in patients with type 2 diabetes, which led to studies on its efficacy for the treatment of obesity in the absence of type 2 diabetes. Remarkably, tirzepatide (15 mg per week) reduced body weight by up to 20.9% after 72 weeks [93], which was associated with reduced waist circumference and an overall improvement in individuals’ clinical profile. The most common adverse events were nausea, vomiting, diarrhoea and constipation. These gastrointestinal side effects appear to be qualitatively similar to those reported in clinical studies of selective GLP-1 receptor agonists. There is also an ongoing cardiovascular outcomes trial of tirzepatide (SURPASS-CVOT) in individuals with type 2 diabetes and cardiovascular disease [91], which, if successful, will broaden the use of tirzepatide to those with manifest or increased risk for CVD, as is the case for GLP-1 receptor agonists [22].

### Triple GIP/GLP-1/glucagon receptor co-agonists

The success of dual receptor agonists for the treatment of type 2 diabetes and obesity in clinical trials, including the approval of one such drug by the FDA and EMA (currently only for type 2 diabetes), has prompted the search for new combinatorial approaches. The synergistic metabolic benefits of simultaneous modulation of glucagon, GLP-1 and GIP receptors through a single molecule were first discovered and validated in 2015 [94]. GLP-1, GIP and glucagon triple agonism reduced body weight and blood glucose levels in diet-induced obese mice to a higher extent than monoagonists and dual GLP-1/GIP receptor agonists [94]. Another triple receptor agonist named SAR441255 was later designed and tested in diet-induced obese mice, confirming the initial discoveries reported in 2015 [95]. This triple receptor agonist exhibited a dose-dependent effect, decreasing body weight by up to 25% after 4 weeks of treatment. The safety, tolerability, pharmacokinetics and pharmacodynamics following single ascending s.c. doses of SAR441255 were studied in lean to overweight individuals and, at the two highest doses tested (80 and 150 ug), fasting blood glucose levels reached a nadir within the first hour [95]. Single doses of SAR441255 up to 150 μg were well tolerated and the most frequent treatment-emergent adverse events were gastrointestinal disorders, as is typical with these compounds.

New triple receptor agonists have recently been designed and have shown a prolonged duration of action, supporting the possibility of once-weekly administration in humans. In one study these drugs decreased body weight in diet-induced obese mice more potently and effectively than could be achieved with semaglutide, tirzepatide, GLP-1/glucagon dual receptor agonists and earlier triple receptor agonists, without affecting glycaemic control [96]. In a separate study, LY343794, another glucagon/GIP/GLP-1 triple receptor agonist, decreased body weight and improved glycaemic control in obese mice [97]. In a Phase I single ascending dose study in healthy participants, LY3437943 showed a similar safety and tolerability profile to that of other incretins and a reduction in body weight up to day 43 after a single dose [97]. This compound was further tested in people with type 2 diabetes and, at week 12, plasma glucose and HbA_1c_ levels decreased significantly from baseline at the highest doses [98]. Whether the clinical success of GLP-1/GIP receptor co-agonists can be surpassed by the triple receptor agonists remains to be shown.

### Other hormone combinatorial approaches

Other gut hormone-based combination pharmacotherapies have been designed and have shown beneficial effects in preclinical models. The strategy employed has been to link GLP-1 or glucagon receptor agonists to nuclear hormones such as oestrogens, thyroid hormones and dexamethasone (reviewed in [64, 96, 99]). For example, a GLP-1/oestradiol conjugate reduced food intake and body weight in different rodent models of the metabolic syndrome, displaying greater efficacy than GLP-1 alone [100]; it also activated the insulin signalling pathway in beta cells, thereby restoring their function and leading to remission of diabetes [101]. In the case of thyroid hormones, T_3_ was conjugated with glucagon to deliver T_3_ specifically to the liver; this resulted in a reduction in body weight and corrected dyslipidaemia in several mouse models of obesity [102]. Finally, dexamethasone was conjugated with GLP-1 and this peptide induced weight loss in obese mice with a greater efficiency than GLP-1 alone [103]. Thus, the use of combination pharmacotherapy involving other molecules in addition to gut hormones seems to be a plausible approach to treating the metabolic syndrome. It should be noted that these unimolecular compounds have been tested only in preclinical models and have not been compared with other dual receptor agonists such as tirzepatide; further studies should elucidate whether their action is superior to that of the clinically tested drugs.

## Outlook and future perspectives

It is 30 years since the first demonstration of the glucose-lowering effects of the gut hormone GLP-1 in people with diabetes [9]. This development has led to the introduction of several GLP-1 receptor agonists and DPP-4 inhibitors on the market [3], which are featured prominently in current recommendations because of their effectiveness, safety and proven cardiovascular benefits [33]. GLP-1 receptor agonists have also shown success in other therapeutic areas apart from type 2 diabetes and obesity, in particular for non-alcoholic fatty liver disease [104]. At the same time, we are facing an exciting multifaceted future, with several novel developments on the horizon with the potential to change the clinic setting, including the development of oral GLP-1 receptor agonists and the emerging formulations of GIP receptor agonists and dual and triple receptor agonists, which have shown clear therapeutic potential. The dual GIP/GLP-1 receptor agonist tirzepatide has already been approved and other dual receptor agonists are expected soon. The dual and triple receptor agonist formulations are also expected to undergo cardiovascular outcomes trials.

Other developments include the ending of patents for DPP-4 inhibitors, which began in 2022 for sitagliptin and which will follow for other DPP-4 inhibitors in the coming years. This will result in lower prices, which may result in increased interest in and use of these drugs. The landscape for guidelines and recommendations is also changing, with a higher emphasis on efficacy than before. Hence, in 2022, glucose-lowering efficacy and proven cardiovascular effects were important for the strong positioning of semaglutide, dulaglutide and tirzepatide in guidelines on the management of hyperglycaemia in type 2 diabetes [33], and a similar strong positioning of incretin therapy was seen in guidelines on primary care [39]. We can also expect more guidelines on weight management and management of kidney disease using incretin therapy, and clinical studies may show beneficial effects of the early introduction of combination therapy on the long-term control of blood glucose levels.

In the future, the successful development of small molecule GLP-1 receptor agonists and therapy based on glucagon and other gut hormones may provide a broader arsenal for the treatment of both hyperglycaemia and overweight. At the same time, increased knowledge of differences in drug response depending on phenotype or genetics may pave the way to more individualised therapy; studies on personalisation are now beginning to emerge, with a recent finding that genetic variations in the GLP-1 receptor, and also in arrestin B1, are important for the glycaemic effects of GLP-1 receptor agonists [105]. The future therefore holds promise for gut hormone-based therapy in the context of both the development of novel drugs and their inclusion in recommendations and guidelines.

## Supplementary Information

Below is the link to the electronic supplementary material.

Slideset of figures (PPTX 723 KB)

## Abbreviations

DPP-4: Dipeptidyl peptidase-4
EMA: European Medicines Agency
EPAC: Exchange protein directly activated by cAMP
FDA: Food and Drug Administration
FGF21: Fibroblast growth factor 21
GIP: Glucose-dependent insulinotropic polypeptide
GLP-1: Glucagon-like peptide-1
PKA: Protein kinase A
SGLT-2: Sodium–glucose cotransporter 2

## References

[1] Moore B, Edie ES, Abram JS (1906). On the treatment of diabetes mellitus by acid extract of duodenal mucous membrane. Biochem J.

[2] Ahrén B (2007). GLP-1 – based therapy of type 2 diabetes. GLP-1 mimetics and DPP-IV inhibitors. Curr Diabetes Rep.

[3] Nauck MA, Meier JJ (2019). Are all GLP-1 agonists equal in the treatment of type 2 diabetes?. Eur J Endocrinol.

[4] Bradley CL, McMillin SM, Hwang HY, Sherrill CH. Tirzepatide, the newest medication for type 2 diabetes: a review of the literature and implications for clinical practice. Ann Pharmacother 2022;10600280221134127. 10.1177/10600280221134127 10.1177/10600280221134127 36367094

[5] Müller TD, Finan B, Bloom SR (2019). Glucagon-like peptide 1 (GLP-1). Mol Metab.

[6] De Graaf C, Donnelly D, Wootten D et al (2016) Glucagon-like peptide-1 and its class B G protein-coupled receptors: a long march to therapeutic successes. Pharmacol Rev 68:954–1013. 10.1124/pr.115.011395 10.1124/pr.115.011395 PMC5050443 27630114

[7] Cannon B, Nedergaard J (2011). Nonshivering thermogenesis and its adequate measurement in metabolic studies. J Exp Biol.

[8] Vilsbøll T, Agerso H, Krarup T, Holst JJ (2003). Similar elimination rates of glucagon-like peptide-1 in obese type 2 diabetic patients and healthy subjects. J Clin Endocrinol Metab.

[9] Gutniak M, Ørskov C, Holst JJ, Ahrén B, Efendic S (1992). Antidiabetic effect of glucagon-like peptide-1 (7–36) amide in normal subjects and patients with diabetes mellitus. N Engl J Med.

[10] Zander M, Madsbad S, Madsen JL, Holst JJ (2002). Effect of 6-week course of glucagon-like peptide 1 on glycaemic control, insulin sensitivity, and beta-cell function in type 2 diabetes: a parallel-group study. Lancet.

[11] Mentlein R (2009). Mechanisms underlying the rapid degradation and elimination of the incretin hormones GLP-1 and GIP. Best Pract Res Clin Endocrinol Metab.

[12] Ahrén B (2001). Inhibition of dipeptidyl peptidase-4 (DPP-4): a target to treat type 2 diabetes. Curr Enzyme Inh.

[13] Mojsov S (1992). Structural requirements for biological activity of glucagon-like peptide-I. Int J Pept Prot Res.

[14] Knudsen LB, Lau J (2019). The discovery and development of liraglutide and semaglutide. Front Endocrinol.

[15] Ahrén B, Burke B (2012). Using albumin to improve the therapeutic properties of diabetes treatments. Diabetes Obes Metab.

[16] Glaesner W, Vick AM, Millican R et al (2010) Engineering and characterization of the long-acting glucagon-like peptide-1 analogue LY2189265, an Fc fusion protein. Diabet Metab Res Rev 26:287–296. 10.1002/dmrr.1080 10.1002/dmrr.1080 20503261

[17] DeYoung MB, MacConell L, Sarin V, Trautmann M, Herbert P (2011) Encapsulation of exenatide in poly-(D,L-lactide-co-glycolide) microspheres produced an investigational long-acting once-weekly formulation for type 2 diabetes. Diabet Technol Ther 13:1145–1154. 10.1089/dia.2011.0050 10.1089/dia.2011.0050 PMC3202891 21751887

[18] Göke R, Fehmann HC, Linn T (1993). Exendin-4 is a high potency agonist and truncated exendin-(9–39)-amide an antagonist at the glucagon-like peptide 1-(7–36)-amide receptor of insulin-secreting beta-cells. J Biol Chem.

[19] Furman BL (2012). The development of Byetta (exenatide) from the venom of the Gila monster as an anti-diabetic agent. Toxicon.

[20] Bolli GB, Owens DR (2014). Lixisenatide, a novel GLP-1 receptor agonist; efficacy, safety and clinical implications for type 2 diabetes mellitus. Diabet Obes Metab.

[21] Yoon KH, Kang J, Kwon SC et al (2020) Pharmacokinetic and dose-finding studies on efpeglenatide in patients with type 2 diabetes. Diabet Obes Metab 22:1292–1301. 10.1111/dom.14032 10.1111/dom.14032 PMC7383501 32175655

[22] Sattar N, Lee M, Kristensen SL (2021). Cardiovascular, mortality, and kidney outcomes with GLP-1 receptor agonists in patients with type 2 diabetes: a systematic review and meta-analysis of randomised trials. Lancet Diabet Endocrinol.

[23] Bode B (2011). An overview of the pharmacokinetics, efficacy and safety of liraglutide. Diabet Res Clin Pract.

[24] Hall S, Isaacs D, Clements JN (2017). Pharmacokinetics and clinical implications of semaglutide: a new glucagonlike peptide (GLP)-1 receptor agonist. Clin Pharmacokinet.

[25] Jimenez-Solem E, Rasmussen MH, Christensen M, Knop FK (2010). Dulaglutide, a long-acting GLP-1 analog fused with an Fc antibody fragment for the potential treatment of type 2 diabetes. Curr Opin Mol Ther.

[26] Onge EL, Miller SA (2010). Albiglutide: a new GLP-1 analog for the treatment of type 2 diabetes. Exp Opin Biol Ther.

[27] Brønden A, Knop FK, Christensen MB (2017). Clinical pharmacokinetics and pharmacodynamics of albiglutide. Clin Pharmacokinet.

[28] Buckley ST, Bækdal TA, Vegge A (2018). Transcellular stomach absorption of a derivatized glucagon-like peptide-1 receptor agonist. Sci Transl Med.

[29] Granhall C, Donsmark M, Blicher TM (2019). Safety and pharmacokinetics of single and multiple ascending doses of the novel oral human GLP-1 analogue, oral semaglutide, in healthy subjects and subjects with type 2 diabetes. Clin Pharmacokinet.

[30] Theti TK, Pratley R, Meier JJ (2020). Efficacy, safety and cardiovascular outcomes of once-daily oral semaglutide in patients with type 2 diabetes: the PIONEER programme. Diabet Obes Metab.

[31] Meier JJ (2021). Efficacy of semaglutide in a subcutaneous and an oral formulation. Front Endocrinol.

[32] Farngren J, Ahrén B (2019). Incretin-based medications (GLP-1 receptor agonists, DPP-4 inhibitors) as a means to avoid hypoglycaemic episodes. Metabolism.

[33] Davies MJ, Aroda VR, Collins BS (2022). Management of hyperglycaemia in type 2 diabetes, 2022. A consensus report by the American Diabetes Association (ADA) and the European Association for the Study of Diabetes (EASD). Diabetologia.

[34] Holst JJ, Deacon CF (1998) Inhibition of the activity of dipeptidyl-peptidase IV as a treatment for type 2 diabetes. Diabetes 47:1663–1670. 10.2337/diabetes.47.11.1663 10.2337/diabetes.47.11.1663 9792533

[35] Ahrén B, Foley JE (2016). Improved glucose regulation in type 2 diabetic patients with DPP-4 inhibitors: focus on alpha and beta cell function and lipid metabolism. Diabetologia.

[36] Ahrén B, Simonsson E, Larsson H (2002). Inhibition of dipeptidyl peptidase IV improves metabolic control over a 4 week study period in type 2 diabetes. Diabetes Care.

[37] Ahrén B (2019). DPP-4 inhibition and the path to clinical proof. Front Endocrinol.

[38] Orime K, Terauchi Y (2020). Efficacy and safety of saxagliptin for the treatment of type 2 diabetes mellitus. Exp Opin Pharmacother.

[39] Subrahmanyan NA, Koshy RM, Jacob K, Pappachan JM (2021). Efficacy and cardiovascular safety of DPP-4 inhibitors. Curr Drug Saf.

[40] Seidu S, Brunton S, Harris SB (2022). 2022 update to the position statement by Primary Care Diabetes Europe: a disease state approach to the pharmacological management of type 2 diabetes in primary care. Prim Care Diabetes.

[41] Ma H, Huang W, Wang X (2020). Structural insights into the activation of GLP-1R by a small molecule agonist. Cell Res.

[42] Kawai T, Sun B, Yoshino H (2020). Structural basis for GLP-1 receptor activation by LY3502970, an orally active nonpeptide agonist. Proc Natl Acad Sci USA.

[43] Saxena AR, Gorman DN, Esquejo RM (2021). Danuglipron (PF-06882961) in type 2 diabetes: a randomized placebo-controlled multiple ascending-dose phase 1 trial. Nat Med.

[44] Alsalim W, Lindgren O, Ahrén B (2023). GIP and GLP-1 secretion in humans – characteristics and regulation. J Diabet Invest.

[45] Gribble FM, Reimann F (2021). Metabolic messengers: glucagon-like peptide-1. Nat Metab.

[46] Jakubowicz D, Froy O, Ahrén B (2014). Incretin, insulinotropic and glucose-lowering effects of whey protein pre-load in type 2 diabetes: a randomized clinical trial. Diabetologia.

[47] Meek CL, Lewis HB, Vergese B, Park A, Reimann F, Gribble F (2016). The effect of encapsulated glutamine on gut peptide secretion in human volunteers. Peptides.

[48] Zhang X, Wall M, Sui Z (2017). Discovery of orally efficacious tetrahydrobenzimidazoles as TGR5 agonists for type 2 diabetes. ACS Med Chem Lett.

[49] Caruso I, Marrano N, Biondi G (2023). Glucagon in type 2 diabetes: friend or foe?. Diabet Metab Res Rev.

[50] Scheen AJ, Lefebvre PJ (2023). Glucagon, from past to present: a century of intensive research and controversies. Lancet Diabet Endocrinol.

[51] Guzman CB, Zhang XM, Liu R (2017). Treatment with LY2409021, a glucagon receptor antagonist, increases liver fat in patients with type 2 diabetes. Diabet Obes Metab.

[52] Liang Y, Osborne MC, Monia BP (2004). Reduction in glucagon receptor expression by an antisense oligonucleotide ameliorates diabetic syndrome in db/db mice. Diabetes.

[53] Dresler CM, Fortner JG, McDermott K, Najorunas DR (1991). Metabolic consequences of (regional) total pancreatectomy. Ann Surg.

[54] Longuet C, Sinclair EM, Maida A (2008). The glucagon receptor is required for the adaptive metabolic response to fasting. Cell Metab.

[55] Geary N, Smith GP (1983). Selective hepatic vagotomy blocks pancreatic glucagon’s satiety effect. Physiol Behav.

[56] Quinones M, Al-Massadi OA, Gallego R (2015). Hypothalamic CaMKKß mediates glucagon anorectic effect and its diet-induced resistance. Mol Metab.

[57] Davidson IWF, Salter JM, Best CH (1960). The effect of glucagon on the metabolic rate of rats. Am J Clin Nutr.

[58] Nair KS (1987). Hyperglucagonemia increases resting metabolic rate in man during insulin deficiency. J Clin Endocrinol Metab.

[59] Whytock KL, Carnero EA, Vega RB (2021). Prolonged glucagon infusion does not affect energy expenditure in individuals with overweight/obesity: a randomized trial. Obesity.

[60] Kleinert M, Sachs S, Habegger KM, Hofmann SM, Müller TD (2019). Glucagon regulation of energy expenditure. Int J Mol Sci.

[61] Müller TD, Finan B, Clemmensen C, DiMarchi RD, Tschöp MH (2017). The new biology and pharmacology of glucagon. Physiol Rev.

[62] Habegger KM, Stemmer K, Cheng C (2013). Fibroblast growth factor 21 mediates specific glucagon actions. Diabetes.

[63] Campbell JE, Ussher JR, Mulvihill EE (2016). TGF_1_ links GIPR signaling to the control of beta cell function and survival. Nat Med.

[64] Müller TD, Clemmensen C, Finan B, DiMarchi RD, Tschöp MH (2018). Anti-obesity therapy: from rainbow pills to polyagonists. Pharmacol Rev.

[65] Althage MC, Ford EL, Wang S, Tso P, Polonsky KS, Wice BM (2008). Targeted ablation of glucose-dependent insulinotropic polypeptide-producing cells in transgenic mice reduces obesity and insulin resistance induced by a high-fat diet. J Biol Chem.

[66] Miyawaki K, Yamada Y, Ban N (2002). Inhibition of gastric inhibitory polypeptide signaling prevents obesity. Nat Med.

[67] Holst JJ, Windeløv JA, Boer GA (2016). Searching for the physiological role of glucose-dependent insulinotropic polypeptide. J Diabet Invest.

[68] Kim SJ, Nian C, Karunakaran S, Clee SM, Isales CM, McIntosch CH (2012). GIP-overexpressing mice demonstrate reduced diet-induced obesity and steatosis, and improved glucose homeostasis. PLos One.

[69] Martin CMA, Irwin N, Flatt PR, Gault VA (2013). A novel acylated form of (d-Ala(2))GIP with improved antidiabetic potential, lacking effect on body fat stores. Biochim Biophys Acta.

[70] Zhang Q, Delessa CT, Augustin R (2021). The glucose-dependent insulinotropic polypeptide (GIP) regulates body weight and food intake via CNS-GIPR signaling. Cell Metab.

[71] Adriaenssens AE, Biggs EK, Darwish T (2019). Glucose-dependent insulinotropic polypeptide receptor-expressing cells in the hypothalamus regulate food intake. Cell Metab.

[72] Killion EA, Lu SC, Fort M, Yamada Y, Véniant MM, Lloyd DJ (2020). Glucose-dependent insulinotropic polypeptide receptor therapies for the treatment of obesity, do agonists = antagonists?. Endocr Rev.

[73] Miroz PA, Finan B, Gelfanov V (2019). Optimized GIP analogs promote body weight lowering in mice through GIPR agonism not antagonism. Mol Metab.

[74] Finan B, Ma T, Ottaway N (2013). Unimolecular dual incretins maximize metabolic benefits in rodents, monkeys, and humans. Sci Transl Med.

[75] Pocai A (2013). Action and therapeutic potential of oxyntomodulin. Mol Metab.

[76] Pocai A, Carrington PE, Adams JR (2009). Glucagon-like peptide-1/glucagon receptor dual agonism reverses obesity in mice. Diabetes.

[77] Day JW, Ottaway N, Patterson JT (2009). A new glucagon and GLP-1 co-agonist eliminates obesity in rodents. Nat Chem Biol.

[78] Corbin KD, Carnero EA, Allerton TD (2023). Glucagon-like peptide-1/glucagon receptor agonism associates with reduced metabolic adaptation and higher oxidation: a randomized trial. Obesity.

[79] Ambery P, Parker VE, Sumvoll M (2018). MEDI0382, a GLP-1 and glucagon receptor dual agonist, in obese or overweight patients with type 2 diabetes: a randomized, controlled, double-blind, ascending dose and phase 2A study. Lancet.

[80] Jepsen MM, Christensen MB (2021). Emerging glucagon-like peptide 1 receptor agonists for the treatment of obesity. Exp Opin Emerg Drugs.

[81] De Block CEM, Drinck E, Verhaegen A, van Gaal LF (2022). Efficacy and safety of high-dose glucagon-like peptide-1, glucagon-like peptide-1/glucose-dependent insulinotropic peptide, and glucagon-like peptide-1/glucagon receptor agonists in type 2 diabetes. Diabet Obes Metab.

[82] Ambery PD, Klammt S, Posch MG (2018). MEDI0382, a GLP-1/glucagon receptor dual agonist, meets safety and tolerability endpoints in a single-dose, healthy-subjects, randomized, phase 1 study. Br J Pharmacol.

[83] Parker VER, Robertson D, Wang T (2020). Efficacy, safety, and mechanistic insights of cotadutide, a dual receptor glucagon-like peptide-1 and glucagon agonist. J Clin Endocrinol Metab.

[84] Marcondes-de-Castro I, Oliveira TF, Spezani R et al (2023) Cotadutide effect in liver and adipose tissue in obese mice. J Mol Endocrinol 70:e220168. 10.1530/JME-22-0168 10.1530/JME-22-0168 36753306

[85] Irwin N, Hunter K, Frizzell N, Flatt PR (2009). Antidiabetic effects of subchronic activation of the GIP receptor alone and in combination with background exendin-4 therapy in high fat fed mice. Regul Pept.

[86] Gault VA, Kerr BD, Harriott P, Flatt PR (2011). Administration of an acylated GLP-1 and GIP preparation provides added beneficial glucose-lowering and insulinotropic actions over single incretins in mice with type 2 diabetes and obesity. Clin Sci.

[87] Nørregaard PK, Deryabina MA, Shelton PT (2018). A novel GIP analogue, ZP4165, enhances glucagon-like peptide-1-induced body weight loss and improves glycaemic control in rodents. Diabet Obes Metab.

[88] Coskun T, Sloop KW, Loghin C (2018). LY3298176, a novel dual GIP and GLP-1 receptor agonist for the treatment of type 2 diabetes mellitus: from discovery to clinical proof of concept. Mol Metab.

[89] Samms RJ, Christe ME, Collins KA (2021). GIPR agonism mediates weight-independent insulin sensitization by tirzepatide in obese mice. J Clin Invest.

[90] Nauck MA, D’Alessio DA (2022). Tirzepatide, a dual GIP/GLP-1 receptor co-agonist for the treatment of type 2 diabetes with unmatched effectiveness regarding glycaemic control and body weight reduction. Cardiovasc Diabetol.

[91] Sinha R, Rapamargaritis D, Sargeant JA, Davies MJ (2023). Efficacy and safety of tirzepatide in type 2 diabetes and obesity management. J Obes Metab Syndr.

[92] De Block C, Bailey C, Wysham C, Hemmingway A, Allen SE, Peleshok J (2023). Tirzepatide for the treatment of adults with type 2 diabetes: an endocrine perspective. Diabet Obes Metab.

[93] Jastreboff AM, Aronne LJ, Ahmad N (2022). Tirzepatide once weekly for the treatment of obesity. N Engl J Med.

[94] Finan B, Yang B, Ottaway N (2015). A rationally designed monomeric peptide triagonist corrects obesity and diabetes in rodents. Nat Med.

[95] Bossart M, Wagner M, Elvert R (2022). Effects on weight loss and glycemic control with SAR441255, a potent unimolecular peptide GLP-1/GIP/GCG receptor triagonist. Cell Metab.

[96] Knerr PJ, Mowery SA, Douros JD (2022). Next generation GLP-1/GIP/glucagon triple agonists normalize body weight in obese mice. Mol Metab.

[97] Coskun T, Urva S, Roell WC (2022). LY3437943, a novel triple glucagon, GIP, and GLP-1 receptor agonist for glycemic control and weight loss: from discovery to clinical proof of concept. Cell Metab.

[98] Urva S, Coskun T, Loh MT (2022). LY3437943, a novel triple GIP, GLP-1, and glucagon receptor agonist in people with type 2 diabetes: a phase 1b, multicenter, double-blind, placebo-controlled, randomised, multiple-ascending dose trial. Lancet.

[99] Clemmensen C, Finan B, Müller TD, DiMarchi RD, Tschöp MH, Hofmann SM (2019). Emerging hormonal-based combination pharmacotherapies for the treatment of metabolic diseases. Nat Rev Endocrinol.

[100] Finan B, Yang B, Ottaway N (2012). Targeted estrogen delivery reverses the metabolic syndrome. Nat Med.

[101] Sachs S, Bastidas-Ponce A, Tritschler S (2020). Targeted pharmacological therapy restores ß-cell function for diabetes remission. Nat Metab.

[102] Finan B, Clemmensen C, Zhu Z (2016). Chemical hybridization of glucagon and thyroid hormone optimizes therapeutic impact for metabolic disease. Cell.

[103] Quarta C, Clemmensen C, Zhu Z (2017). Molecular integration of incretin and glucocorticoid action reverses immunometabolic dysfunction and obesity. Cell Metab.

[104] Nevola R, Epifani R, Imbriani S (2023). GLP-1 receptor agonists in non-alcoholic fatty liver disease: current evidence and future perspectives. Int J Mol Sci.

[105] Dawed AY, Mari A, Brown A (2023). Pharmacogenomics of GLP-1 receptor agonists: a genome-wide analysis of observational data and large randomized controlled trials. Lancet Diabet Endocrinol.

