# Supplementary material for: Gut hormone-based pharmacology: novel formulations and future possibilities for metabolic disease therapy
Source: Diabetologia. 2023 May 20;66(10):1796–808. doi: 10.1007/s00125-023-05929-0 (PMC10474213; doi:10.1007/s00125-023-05929-0)
Supplement: Supplementary file 1 — Slideset of figures (PPTX 723 KB) [file 125_2023_5929_MOESM1_ESM.pptx]

## Slide 1
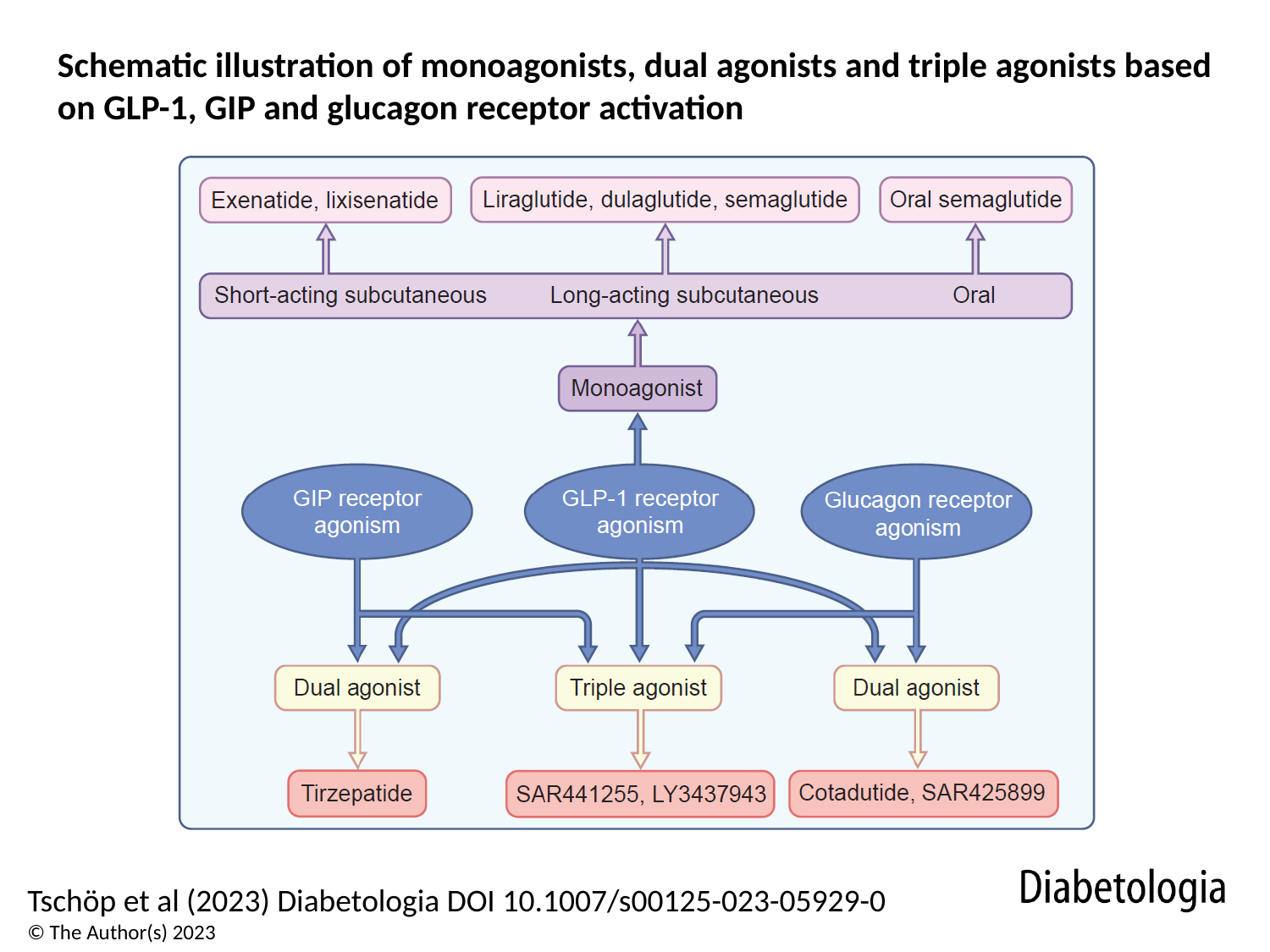

Schematic illustration of monoagonists, dual agonists and triple agonists based on GLP-1, GIP and glucagon receptor activation
Tschöp et al (2023) Diabetologia DOI 10.1007/s00125-023-05929-0
© The Author(s) 2023

## Slide 2
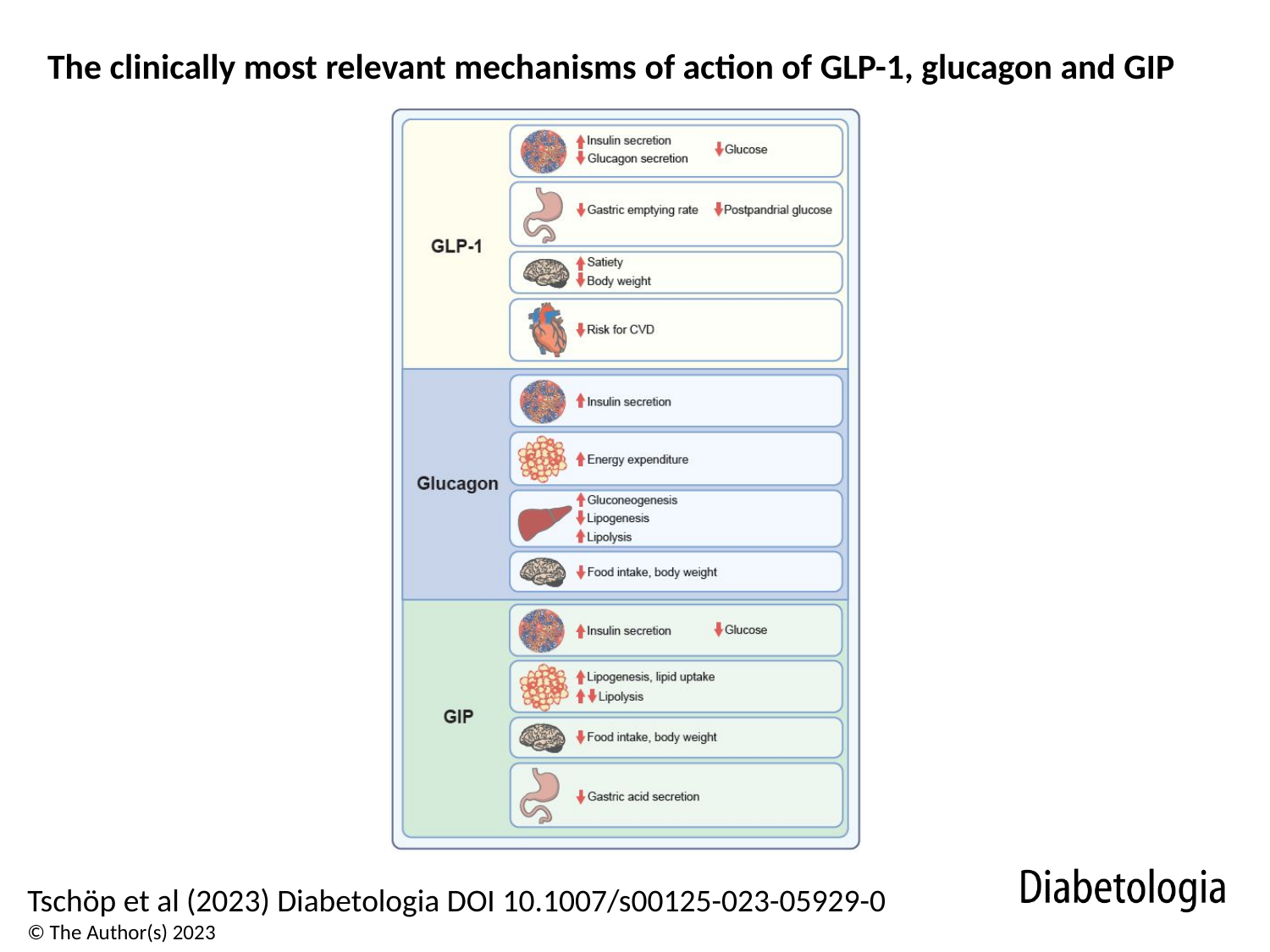

The clinically most relevant mechanisms of action of GLP-1, glucagon and GIP
Tschöp et al (2023) Diabetologia DOI 10.1007/s00125-023-05929-0
© The Author(s) 2023

## Slide 3
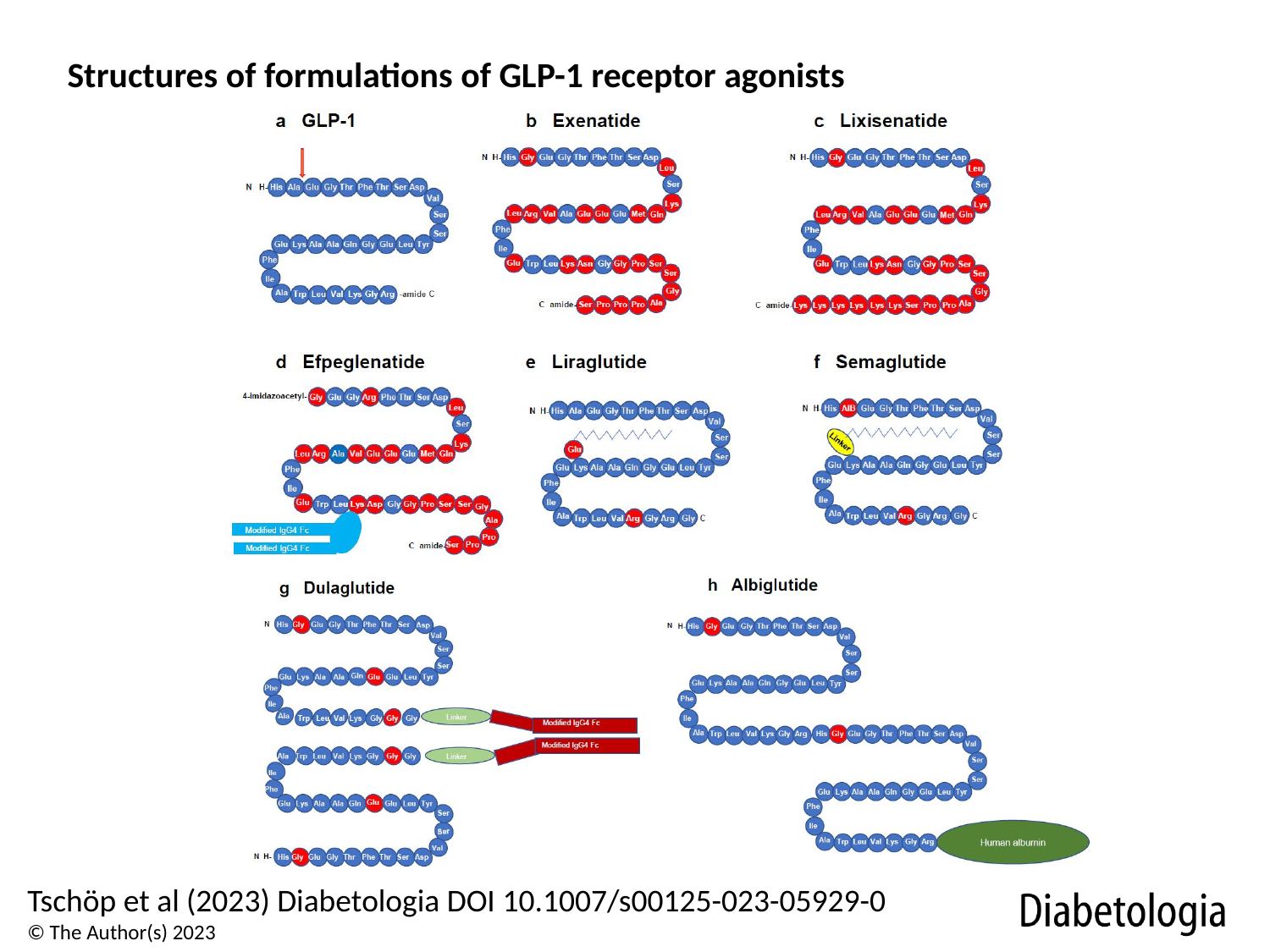

Structures of formulations of GLP-1 receptor agonists
Tschöp et al (2023) Diabetologia DOI 10.1007/s00125-023-05929-0
© The Author(s) 2023

## Slide 4
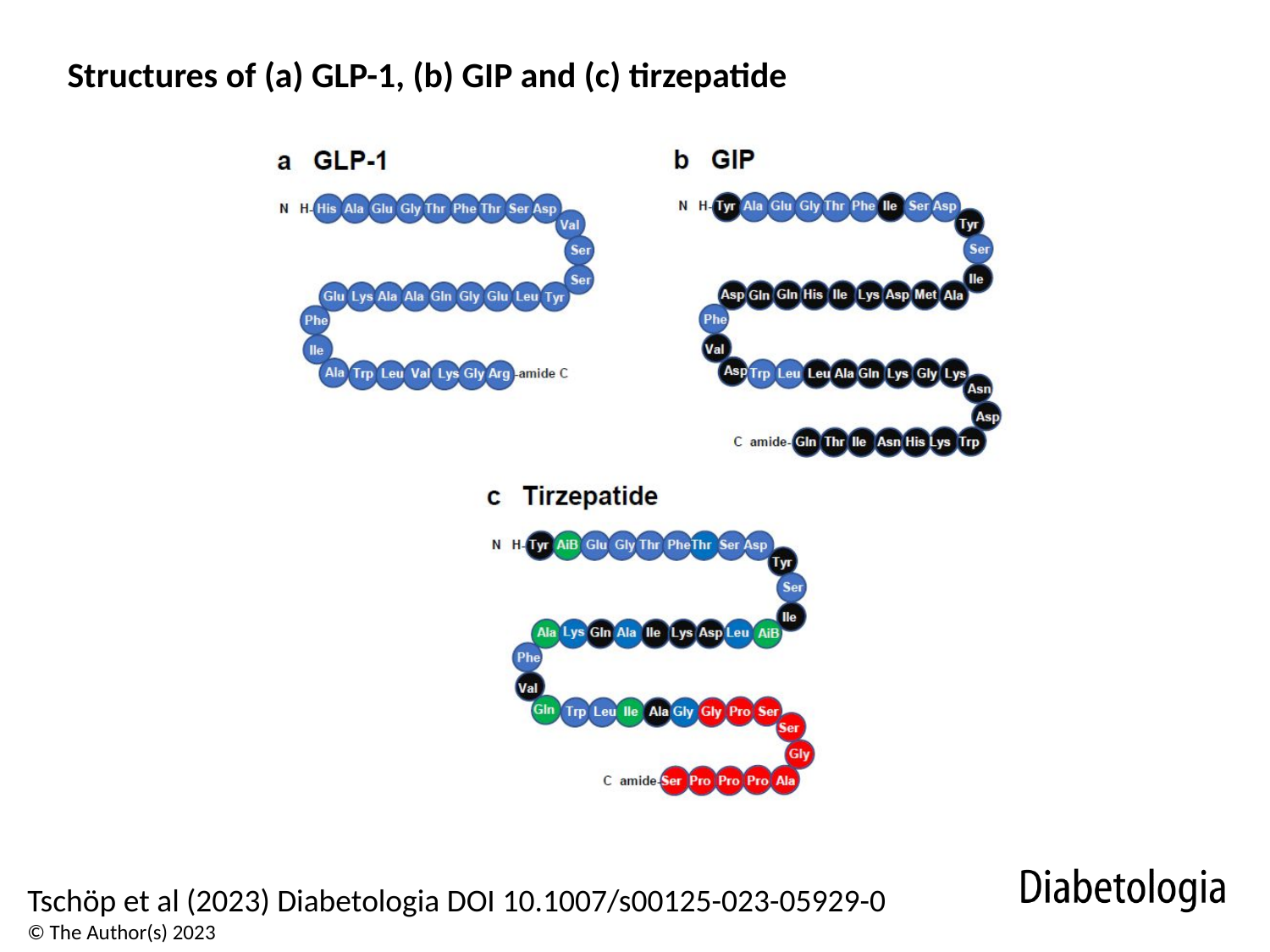

Structures of (a) GLP-1, (b) GIP and (c) tirzepatide
Tschöp et al (2023) Diabetologia DOI 10.1007/s00125-023-05929-0
© The Author(s) 2023
